# From welcome culture to welcome limits? Uncovering preference changes over time for sheltering refugees in Germany

**DOI:** 10.1371/journal.pone.0199923

**Published:** 2018-08-01

**Authors:** Ulf Liebe, Jürgen Meyerhoff, Maarten Kroesen, Caspar Chorus, Klaus Glenk

**Affiliations:** 1 Department of Sociology, University of Warwick, Coventry, United Kingdom; 2 Institute for Landscape and Environmental Planning, Technische Universität Berlin, Berlin, Germany; 3 Faculty of Technology, Policy and Management (TPM), Delft University of Technology, Delft, The Netherlands; 4 Land Economy, Environment & Society Group, SRUC, Edinburgh, United Kingdom; Brown University, UNITED STATES

## Abstract

Europe recently experienced a large influx of refugees, spurring much public debate about the admission and integration of refugees and migrants into society. Previous research based on cross-sectional data found that European citizens generally favour asylum seekers with high employability, severe vulnerabilities, and Christians over Muslims. These preferences and attitudes were found to be homogeneous across countries and socio-demographic groups. Here, we do not study the general acceptance of asylum seekers, but the acceptance of refugee and migrant homes in citizens’ vicinity and how it changes over time. Based on a repeated stated choice experiment on preferences for refugee and migrant homes, we show that the initially promoted “welcome culture” towards refugees in Germany was not reflected in the views of a majority of a sample of German citizens who rather disapproved refugee homes in their vicinity. Their preferences have not changed between November 2015, the peak of “welcome culture,” and November 2016, after political debates, media reporting and public discourse had shifted towards limiting admission of immigrants. A minority of one fifth of the sample population, who were initially rather approving of refugee and migrant homes being established in their vicinity, were more likely to change their preferences towards a rather disapproving position in 2016. Experience of contact with refugees and migrants, higher education, and general pro-immigration attitudes explain acceptance of refugee and migrant homes as well as preference stability over time. Country of origin and religion of refugees and migrants are considered less important than decent housing conditions and whether refugee and migrants arrive as families or single persons. In this respect our results highlight the importance of humanitarian aspects of sheltering and integration of refugees and other migrants into society.

## Introduction

The term “welcome culture” (Willkommenskultur) is characteristic of Germany’s initial public discourse and official response to the hundreds of thousands of refugees arriving to the country in the wake of the Syrian civil war in 2015. It stands for a call for accepting those in need and encouraging support of the civil society in the integration of refugees. Many observers agree, however, that Germany’s political and societal perspective on admitting refugees has since shifted [1, 2, 3]. This shift is reflected in heated political discussions before and during the 2017 general elections about “refugee ceilings” and the “refugee crisis,” two terms which are now established in politics, media and everyday language.

Close to one million mostly Syrian refugees and asylum seekers entered Germany in the second half of 2015 [4]. In September 2015 the first big wave of refugees–people with a well-founded fear of persecution in their country of nationality or habitual residence–and migrants–people who voluntarily leave their home country in pursuit of a better live–arrived in what seemed to be a predominantly friendly, welcoming climate. Many German citizens were willing to help directly or indirectly, and some accommodated refugees in their homes [5]. Positive views expressed at that time were also related to expectations of refugees filling gaps in the labour market over time [6]. However, the persistent arrival of refugees and migrants has since prompted debates about the number of refugees that Germany can manage to shelter, and about competition between refugees and German citizens for scarce public resources [7]. Tensions have emerged especially about the supply and management of refugee and migrant homes. In several cases the police have had to protect refugees and migrants as they were moved to their temporary accommodation [8]. Prospective refugee homes were set on fire, successfully preventing their completion [1]. Protests against accommodating refugees and migrants also occurred in better-off neighbourhoods [9].

Against a background of rising political and societal tensions in Germany, as elsewhere, little to nothing is known about the mechanisms of opinion formation and preference changes concerning the acceptance of refugee and migrant homes. Previous research [10] investigated attitudes towards types of asylum seekers in Europe at one point in time, March 2016, and found commonalities across 15 European countries: citizens favoured asylum seekers with high employability, consistent asylum testimonies and severe vulnerabilities, and Christians over Muslims. These attitudes did not differ depending on citizens’ political ideology, age, education and income.

This study specifically explores preferences for refugee and migrant homes in one’s neighbourhood. Simple survey questions on general attitudes towards immigration as frequently implemented in opinion polls [11, 12, 13] can be considered to be a relatively weak instrument to measuring preferences regarding immigrant admission and distribution. Instead, providing survey respondents with concrete choice situations that recognise multiple dimensions of decision-making allows for a more specific and hence stronger measurement of preferences, especially if there is a realistic chance that respondents are directly affected by the decision context [10, 14, 15]. This is the case in our study, because preferences are measured in the context of establishing refugee and migrant homes in the neighbourhoods and city districts where survey respondents reside. Furthermore, this study investigates transitions in preferences over time. Compared to static polls or surveys, a dynamic perspective takes into account potential changes in preferences and acknowledges that public opinion is known to be susceptible to change. Additionally, it allows studying potential factors underlying such transitions.

## Data and methods

Based on responses to a stated choice experiment [16, 17, 18], a multifactorial survey considering different attributes of refugee and migrant homes, we firstly analyse individuals’ preferences for establishing different types of new refugee and migrant homes in their vicinity. By administrating the exact same survey to the same individuals one year later we secondly assess how these preferences may have changed over the course of a year, during which overwhelming media attention was devoted to the topic of refugees in the study area.

Specifically, the data were collected in collaboration with the survey organization LINK(FORSA), which manages an online panel that is recruited by telephone (as opposed to using an opt-in online access panel) to ensure that panel members are from representatively selected households in Germany. In November 2015, 5,128 panel members were invited to take part in the survey; 861 completed the survey, 194 dropped out of the survey, and 996 were screened out because of quota restrictions related to gender, age, education, and smartphone use. This results in a response rate of 21% (RR1 rate, AAPOR) [19]. All 861 respondents of the first survey in November 2015 were invited for a second interview in November 2016; 573 (67%) took part, yielding an attrition rate of 33%. In the following analysis we consider all n = 418 respondents who did not have any missing values for the variables considered in this study. This set-up can be characterized as a test-retest design.

Our study population is close to being representative for the German population with respect to sex (49% women in the sample versus 51% in the general population) and age (mean of 43 years in the sample versus 44 years in the general population) but not so with respect to education (Table A in S1 File). While 54% in our sample have higher education (at least 12 years of education), this share is much lower in the general population (29%). This creates a sample bias with respect to education. However, since our results presented below suggest that education has a positive effect on preferences for refugee and migrant homes as well as on preference stability over time, it is likely that in a sample with greater representation of less educated individuals one would find a higher share of negative preferences and attitudes towards refugee and migrant homes, further strengthening our overall conclusion that claims about the “welcome culture” in German society might have been exaggerated.

Further analysis shows that age is the only characteristic with a statistically significant (and positive) effect on retest participation, while variables such as general pro-immigration attitudes do not significantly affect retest-participation; this strengthens the validity of our analyses (Table B in S1 File, also Table D in S1 File).

All respondents participated in a repeated stated choice experiment on the acceptance of refugee and migrant homes in their vicinity. The decision context of the need for new homes for refugees and migrants and the attributes of these homes were carefully chosen to reflect the actual situation faced by communities across Germany at the time of the survey. Respondents were asked to imagine that new refugee and migrant homes that would be inhabited for at least three years had to be built in the vicinity of their place of residence. Respondents then faced a series of six choice tasks, each offering three specifications of refugee and migrant homes, which differed in the five attributes (1) main country of origin and religion of the refugees/migrants; (2) number of people; (3) type of housing; (4) distance to respondent’s home; and (5) whether mainly single persons or families would live in the homes (see Tables 1 and 2). Note that the terms refugee and migrant are not in line with definitions according to international law (e.g., refugees are also migrants). However, we clearly defined both terms for respondents (see suppl. material) and used them in the survey because they reflect societal debates in 2015 and were frequently used in media, politics, and society (e.g., the German news program “Tagesschau,” [20]). With respect to country of origin and religion, we selected two countries (Syria and Serbia) that are within the top-ten countries in terms of numbers of asylum seekers being registered in Germany in 2015, and two countries (Nigeria and India) that are less common as countries of origin of asylum seekers in Germany [21]. The countries presented in the choice sets were also meant to reflect different degrees of cultural distance to Germany. Another important selection criterion was that all of these countries have a sizeable population of Christians, which forms a common reference regarding religion. All other attributes reflect public debates in 2015 about how many immigrants “neighborhoods,” city districts and small towns can accommodate, whether families are preferred over single persons, what decent housing conditions are, and how close new homes should be built to residents. Because it was evident in 2015 that establishing new homes for refugees was the only option, an explicit opt-out alternative was not offered. However, respondents could decide not to answer the choice tasks, and move ahead in the questionnaire. Each respondent answered the exact same six choice tasks in both surveys, with one year in between them.

**Table 1:** Overview of attributes in the stated choice experiment.

Attribute	Levels
Main country of origin and religion	Syria (Muslims), Syria (Christians), Serbia (Serbian-Orthodox), Serbia (Christian), Nigeria (Muslims), Nigeria (Christian) or India (Hindus), India (Christians)
Number of persons	12, 32, 84, 125, 220, 350 persons
Mainly families or single persons	Mainly families with children or single persons
Type of home	Empty, renovated house; container building; an empty large building (e.g., building center or hospital) or an existing multi-purpose hall (e.g., gymnasium)
Distance to respondent’s house / flat	500m, 1000m, 1700m or 2500m

**Table 2:** Example of a choice task in the stated choice experiment.

	Refugee Home	Refugee and Migrant Home	Migrant Home
Main country of origin and religion	Syria (Muslims)	Syria (Christians)	Serbia (Serbian-Orthodox)
Number of persons	220	350	12
Mainly families or single persons	Families with children	Families with children	Single persons
Type of home	Container building	Renovated house	Multi-purpose hall
Distance form your house/flat	1700m	2500m	500m
I choose …	□	□	□

Notes: The question in each choice task was worded as follows: “The establishment of a home for refugees and/or migrants in the area where you live could be as described in the choice sets. Please choose the best alternative for you. (Please assume that all alternatives would be feasible in your place of residence.).”

To ensure that respondents understood the choice tasks as intended by the researchers, we employed five think-aloud cognitive pretesting interviews [22, 23] and n = 99 pretest interviews of the web survey where we explicitly asked for comments on the questionnaire. We made changes on the survey instrument following these pretest interviews and believe that, on average, respondents understood the information and tasks given as intended. As there was no indication that respondents perceived attribute combinations in certain alternatives as implausible, we decided not to exclude specific attribute combinations, which would have been detrimental to the statistical efficiency of the design. Further, in the main survey after completing all choice tasks, we asked respondents in an open question to describe how they made their choices. These answers reveal that the respondents did not have difficulties to answer the choice sets. They provided clear answers on how they approached the choice tasks, and which attributes they considered most when making their decisions. Further, confirming the pretest interviews, we did not receive feedback by the respondents of the main survey about perceptions of implausible choice tasks, which might have led to biased responses similar to ‘protest’ responses in public good valuation using stated choice experiments [24].

Our study considers several factors which might explain initial preferences and changes in preferences towards refugee and migrant homes. Following the contact hypothesis of interethnic relations [25, 26, 27], contact between refugees/migrants and natives should foster pro-immigrant preferences and weaken prejudices and stereotypes. It follows that contact between refugees and natives should also increase the acceptance of refugee homes as well as preference stability over time, because individual judgments are less ambiguous. This should also hold true for higher educated individuals, who are expected to obtain more (political) information which stabilizes their opinions, attitudes and preferences, even if the social context changes [28, 29]. Higher/lower acceptance and relatively stable preferences are also expected for those individuals who hold very positive/very negative general attitudes towards immigration. Such general attitudes are manifest attitudes and hence their effect on specific preferences and behaviour should be less likely to change over time compared to ambiguous general attitudes [29, 30].

To obtain an initial impression of the preferences in the sample as a whole we started with the standard *multinomial logit model*, in which we included respondents from both waves and all attribute levels as described in Table 1 (see Table C in S1 File). Since our interest was not in revealing individuals’ preferences towards a range of specific countries and religions, but was actually focused on preferences towards Muslim and Syrian migrants/refugees, we estimated a second MNL model in which we considered the effect of these particular attribute levels relative to all other attribute levels combined (see Table D in S1 File).

In order to reveal and explain transitions in preferences over time, a method was needed to identify groups of individuals with similar preferences, as this would allow us to model and explain transitions of individuals between the identified groups over time. In order to obtain these homogenous groups with similar preferences, we applied latent class choice modelling [31, 32]. Within the *latent class choice model* it is assumed that people’s choices/preferences are conditional on their membership of an unobserved (latent) class. Individual assignment to these classes is probabilistic, meaning that each individual has a certain probability to belong to each of the latent classes. Six explanatory variables (gender, age, education, pro-immigrant attitude, contact with migrants, shelter near home; see Table A in S1 File) are included in the class membership function. By treating individuals from different waves as different units, we allowed individuals to switch class membership across both waves.

The identification of two classes (see Tables E and F in S1 File) allowed modelling and interpreting transitions of individuals between these classes over time. To this end, a *2-state Markov model* was estimated [33]. Based on modal assignment, each individual in each wave is (deterministically) assigned to one of the two classes, i.e. the class with highest membership probability for that individual. In the Markov model it is assumed that class membership represents a ‘state’ of an individual. In line with the first-order Markov assumption, it is assumed that a person’s state membership at the second point in time (2016) is influenced by his/her state membership at the first point in time (2015). In addition, the six explanatory variables previously included in the class membership function are again included in the model and assumed to predict initial state membership as well as state membership at the second point in time.

### Ethical compliance statement

This study is based on a standard population survey carried out in collaboration with the survey organization LINK(FORSA). Respondents were members of LINK(FORSA’s) access panel for online surveys and survey participation was voluntary. Further, answering each question in the survey was voluntary (i.e. each respondent had the opportunity to not answer a particular question). The survey was conducted in line with the standards/ethics recommended by AAPOR. At the universities, where this study has been conducted, such population surveys do not need specific ethical approval and we therefore did not seek ethic approval in this case.

## Results

Table 3 reports the results of a latent class choice model for 2015, which captures preference heterogeneity by means of classifying individuals, based on their choices made in the stated choice experiment and with a certain probability, into distinct groups (classes) that are homogenous in terms of their preferences. Two classes with considerably different preference patterns emerge, suggesting a strong degree of preference heterogeneity towards migrant and refugee homes in the study population. It is common to label the classes for ease of presentation; that is, to attach a label to each class that is descriptive of the distinct preference pattern within each class. We acknowledge that every label will inevitably represent a simplified and potentially debatable summary of preference patterns that is subjectively defined post-hoc by the researcher. Bearing this in mind, class 1 is denoted ‘rather disapproving’ of refugee and migrant homes in their vicinity, while class 2 is denoted ‘rather approving’ of refugee and migrant homes in their vicinity. Estimated class sizes are 80% for class 1 and 20% for class 2. That is, 80% of respondents have a greater likelihood of adhering to preference patterns as estimated for class 1, and 20% have a greater likelihood of being allocated to class 2.

**Table 3:** Parameter estimates of the 2-class choice model for 2015.

		Class 1		Class 2	
**Class size**		0.797		0.203	
**Attribute**	**Attribute levels**	**Estimate**	**SE**	**Estimate**	**SE**
Intercepts (ref. = refugee and migrant homes)	Refugee homes	0.082	0.045	0.147	0.132
	Migrant homes	**-0.406**	0.051	-0.131	0.234
Main country of origin and religion (ref. = all other)	Syrian	**0.484**	0.062	**0.451**	0.200
	Muslim	**-0.735**	0.061	0.203	0.183
Number of persons (/ 100)	Continuous	**-0.262**	0.023	-0.093	0.064
Family (ref. = single person)	Mainly families	**0.854**	0.045	**0.797**	0.154
Type of home (ref. = a container)	Empty large building	**0.319**	0.067	**2.222**	0.317
	Multi-purpose hall	-0.037	0.060	0.255	0.314
	Renovated house	**0.272**	0.071	**2.761**	0.346
**Class membership (ref. = class 2)**		**Estimate**	**SE**		
Intercept		**9.282**	1.542		
Gender (ref. = male)	Female	0.320	0.288		
Age	Continuous	-0.000	0.010		
Education level	Continuous	**-0.087**	0.040		
Pro-immigrant attitude	Continuous	**-1.999**	0.362		
Contact (ref. = no)	Yes	**-0.811**	0.352		
Shelter near home (ref. = no)	Yes	-0.678	0.387		
** **	**Pseudo-R^2^**				
**Class 1**	0.143				
**Class 2**	0.429				
**Overall**	0.226				

SE = standard error

Estimates in bold are significant at p<0.05

Individuals belonging to the “rather approving” class are indifferent between homes for refugees and homes for migrants whereas individuals in the “rather disapproving” class prefer refugee homes over migrant homes. Individuals in the rather disapproving class favour non-Muslim refugees and migrants compared to Muslim refugees and migrants, while members of the rather approving class are indifferent between Muslim and non-Muslim refugees and migrants. In contrast to members of the rather approving class, those in the rather disapproving class have a preference for maximizing the physical distance to refugees or migrants as well as for minimizing the number of refugees or migrants. Members of the rather approving class, however, prefer lower distances between refugee and migrant homes and their own homes and are indifferent to the number of refugees and migrants in the home.

Apart from these significant and sizable differences in preferences, the classes also share some common characteristics. Both classes show a preference for better housing conditions (empty large building; renovated house); albeit this preference is stronger in the rather approving class. Common across the two classes, refugees and migrants from Syria are preferred relative to all other countries considered (i.e. India, Nigeria, Serbia); and a preference is observed for families over single persons. Across the whole sample, our analysis shows that decent housing conditions and family migration as opposed to single persons are more important drivers of preferences for refugee or migrant homes than country of origin and religion (Tables H and I in S1 File).

**Fig 1:**
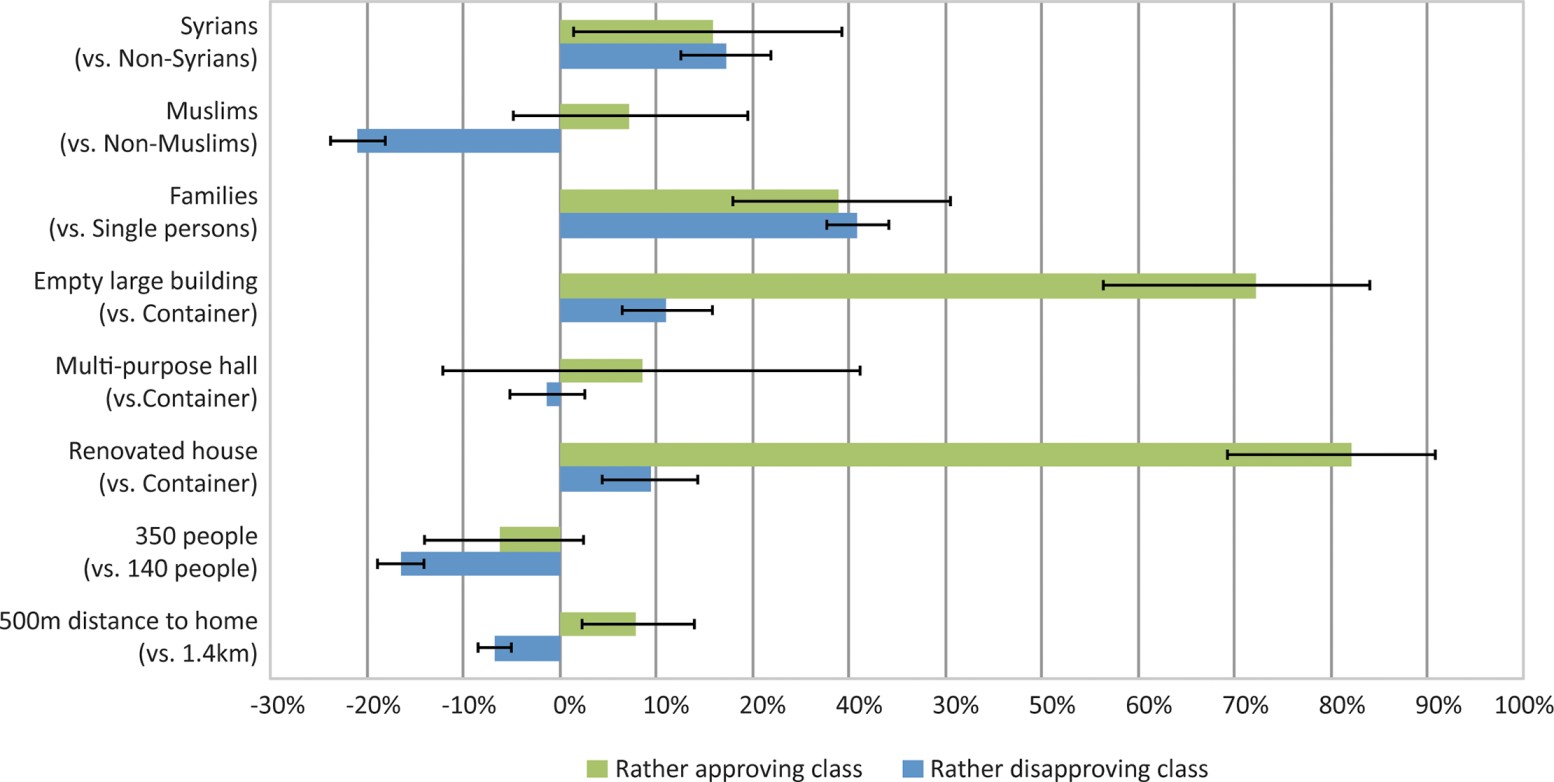
Influence of different characteristics of the refugee/migrant homes on the probability of accepting a home in the vicinity.

Notes: Presented are estimated probabilities based on the 2-class choice model for 2015 as shown in Table 2. Attribute changes are evaluated relative to a reference specification that jointly accommodates refugees and migrants, is a container building, accommodates about 140 people, non-Syrians, non-Muslims, mainly single persons and is located about 1.4 kilometres away from respondents’ home. Error bars represent 95% confidence intervals. Error bars that cross the 0%-line indicate that individuals are indifferent between attribute levels: the attribute effect on choice probabilities is statistically not different from zero. For example, the rather approving class is not significantly more likely to choose a refugee or migrant home if that home hosts Muslims rather than non-Muslims.

To obtain an understanding of differences in effects between both groups, we analyse how changes in the specification of a refugee or migrant home impact on the probability of choosing this home from a choice set containing three homes. Fig 1 shows the corresponding results (also Table G in S1 File). Different specifications are evaluated relative to a reference specification that jointly accommodates refugees and migrants, non-Syrians, non-Muslims, mainly single persons, is a container building, accommodates about 140 people, and is located about 1.4 kilometres away from respondents’ own home. Compared to the reference specification, the probability that a randomly sampled individual prefers a home with particular specification increases by 17% (rather disapproving class) and 16% (rather approving class) if the home accommodates Syrians compared to non-Syrians. It decreases by 21% (rather disapproving class) if the home accommodates Muslims compared to non-Muslims. The probability of choosing a home that is an empty large building is increased by 11% in the rather disapproving class and 72% in the rather approving class. The corresponding figures for a renovated house are an increase of 9% (rather disapproving class) and 82% (rather approving class). Families are associated with an increase of 31% in the rather disapproving class and an increase of 29% in the rather approving class. In the rather disapproving class, a maximum number of 350 people in the homes decreases the choice probability by 17% and a minimum distance of 500 metres to respondents’ homes results in a decrease of 7%. In contrast, proximity (a minimum distance of 500 metres to respondents’ homes) increases the choice probability by 8% in the rather approving class.

What explains membership to the rather disapproving class relative to being in the rather approving class? Of the three factors that are significantly associated with class membership, all are in line with theoretical expectations. That is, those individuals who have been in contact with refugees, who are better educated and who have a more positive attitude towards immigrants in general are less likely to be a member of the rather disapproving class.

Table 4 reports the transition probabilities based on the 2-state Markov model [34] (Table J in S1 File). The results show that a randomly sampled individual who is in the rather disapproving class in 2015 is, with a probability of 90%, very likely to remain in this class in 2016. Preference changes are therefore unlikely for those who are initially disapproving refugee and migrant homes in their vicinity. In contrast, a randomly sampled individual who is in the rather approving class in November 2015 has, with 44%, a relatively high probability to move to the rather disapproving class in November 2016. These individual-level transition probabilities are in line with reported negative shifts in the aggregate opinion on refugees during this time period [35].

**Table 4:** Matrix of transition probabilities from 2015 to 2016.

	State in 2016		
State in 2015	Rather disapproving	Rather approving	Total
Rather disapproving	90%	10%	100%
Rather approving	44%	56%	100%

Which factors explain preference changes over time? The same factors that were influential for pro-refugee-home preferences are found to play a role (Table J in S1 File). Individuals who have been in contact with refugees, who already have a refugee or migrant home in their vicinity, who are better educated and have stronger general pro-immigration attitudes are more likely to shift to (or stay in) the rather approving class, and less likely to stay in (or shift to) the rather disapproving class.

## Discussion and conclusions

Summing up, the preferences of those who are disapproving of refugee and migrant homes–the majority in the sample–are by and large found to be temporally stable. However, the positive preferences for refugee and migrant homes of individuals assigned to the rather approving class in November 2015 are relatively unstable over time. With 20% (in November 2015) this group is a minority in our sample. Our findings, therefore, identify a rather small group in the sample which is prone to preference changes which are most likely affected by public debates and immigration-related events between 2015 and 2016.

Taking the relatively small size of the volatile class into account, our study therefore indicates rather stable preferences for refugee and migrant homes over time across the whole sample. The majority of the study population is rather disapproving of refugees and migrants living in their vicinity, and a decreasing minority has positive preferences towards refugees and migrant homes in their vicinity. This suggests that the “welcome culture” was actually not present in German society to the extent suggested by many media reports and opinion polls, and by its promotion through politicians. Of course, we are not able to measure “welcome culture” and the change thereof directly, but our findings partly complement other (panel) studies that show a decrease in the acceptance of immigrants in Germany between 2015 and 2016 [36]. While in these studies respondents were asked, at a rather general level, about the willingness to give immigrants the right to live in Germany [36], we focused, at a rather specific level, on the acceptance of refugee and migrant homes in citizens’ vicinity. Furthermore, we did not offer an opt-out option in the choice sets, i.e. the possibility to not choose a refugee and migrant home at all. The reason was that it has been clear at the time of the survey that homes have to be constructed. If respondents would make use of such an opt-out option, this would have indicated a negative preference towards refugees and migrants and also strengthen our conclusion regarding “welcome culture”.

A potential explanation for the discrepancy between media reports on “welcome culture,” also based on opinion polls, and our results is the presence of Not-In-My-Back Yard (NIMBY) beliefs [37, 38]. Opinion poll results based on survey questions concerning the *general* acceptance of refugees apparently differ from our results, which are based on responses to choice tasks containing specific types of migrant and refugee homes at the local level, in the vicinity of the respondents’ place of residence.

Our study has several limitations. First, we cannot rule out a hypothetical bias, i.e. differences between stated and actual preferences, as people answered a survey and did not actually place a vote, for example [39]. Yet, a previous study suggests that even in the related and also rather sensitive context of naturalisation, hypothetical bias is found to be limited [40]. Nevertheless, our study could be complemented by revealed preference studies that look on actual behavioural choices in response to the construction of refugee and migrant homes in citizens’ vicinity. For example, do citizens move out or protest in response to the construction of new homes and, if so, does this depend on characteristics of the homes at hand. Second, compared to studies on the general acceptance of immigrants [10, 14, 36], we include less characteristics of immigrants in our experiment and focus more on housing conditions. For example, other studies also refer to the language skills, profession, and employment plans of immigrants [10, 14, 36]. The presence or absence of these attributes can alter effects of the country of origin and religion. However, in our study we find that some of the refugees’ and migrants’ attributes are less important than the housing conditions; i.e., there is no general trend that country of origin and religion is, due to omitted variables, dominating stated preferences. Furthermore, preferences for some attributes such as number of persons might represent a general dislike of housing developments in citizens’ vicinity. We cannot exclude this possibility but answers to open questions following the choice tasks provide no hints that this might be the case, because respondents relate the number of persons explicitly to refugees and migrants. While we used pretesting interviews to test our survey instruments and the experimental tasks, a stronger involvement of the target population in the design of the experiments is recommended for future studies. This prevents potential bias from omitted attributes and ensures that attributes and their characterisation in the choice experiment reflect all important aspects as perceived by people. Third, we do not have a representative sample. While our sample is fairly close to the general population regarding gender and age, there exists an education-related bias towards higher educated individuals. However, we observe a positive correlation between education and preferences in favour of refugee and migrant homes, and it can therefore be assumed that a less biased sample would support our conclusions regarding a change in preferences over time in disfavour of refugee and migrant homes. Finally, we could have opted for another analysis strategy to study preference change over time. The most logical alternative would be to estimate separate (MNL) models for each point in time and compare the differences in the parameter estimates. While such an approach would allow us to examine shifts in aggregate-level preferences towards the particular attributes, it would not allow us to assess preference changes on the level of individuals. Apart from providing more intuitive and interpretable results regarding preference change over time, our methodological approach also allowed us to explore the (individual) correlates of preference change, one of the main objectives of the present study.

Notwithstanding these limitations of and alternatives to our approach, our results raise several points that should be considered for (immigration) policy design in Germany. First, while a minority in our sample does not distinguish between refugees and migrants, a clear majority shows a preference for refugees over migrants. This may reflect a tendency to separate the “deserving” refugee in need for (temporary) care and support from the state from the “undeserving” migrant [41]. This is in line with previous findings on the acceptance of immigrants in Germany and other European countries showing that migrants who immigrate due to better economic opportunities are less accepted than those who immigrate due to persecution [10, 36]. Second, refugees or migrants entering as families are preferred over single persons. Facilitating family reunions of refugees might therefore be valued positively. With respect to single persons, especially an influx of single male immigrants will likely be disapproved if we assume that respondents in our study imagined predominantly male immigrants and believe that single male immigrants are more likely to exhibit deviant behaviour [42], as has been discussed, for example, after the mass sexual assaults on New Year’s Eve in the city of Cologne in 2015/2016 [43]. Third, the country of origin and religion matter with preferences being in favour of non-Muslim refugees from Syria. However, aspects such as families vs. single persons and decent housing conditions are found to be more important. The strong emphasis on origin and religion frequently given in media reports and political statements therefore appears to be unjustified.

Our research provides novel insights in two main dimensions. First, we find heterogeneity in terms of preferences for refugee/migrant homes in the vicinity of citizens’ homes; second, we find preferences have changed over time for a small subset of the study population which initially held positive views. In line with previous research humanitarian aspects are found to be important determinants of preferences for (homes for) refugees and other migrants. In this respect and against a background of persistent immigration in Germany and elsewhere our results add to the evidence [10] supporting an immigration policy that stresses such aspects of sheltering and integrating refugees and other migrants into society.

## Supporting information

S1 File Supporting information. (PDF)
