# Supplementary material for: From welcome culture to welcome limits? Uncovering preference changes over time for sheltering refugees in Germany
Source: PLoS One. 2018 Aug 1;13(8):e0199923. doi: 10.1371/journal.pone.0199923 (PMC6070168; doi:10.1371/journal.pone.0199923)
Supplement: S1 File — (PDF) [file pone.0199923.s001.pdf]

Sample and Variables

Table A provides an overview of sample characteristics which are used in our empirical analyses to explain class membership in the latent class choice model and a 2-state Markov model. We consider all respondents who did not have any missing values for the variables considered in this study.

Table A. Overview on sample characteristics (n = 418).

Variable (year)	Mean	SD	Min	Max
Sex (1 = women, 2015)	0.486	0.50	0	1
Age in years (2015)	42.86	14.25	18	81
Education in years (2015)	12.78	3.67	7	18
General attitude towards immigration (2015)	2.66	0.60	1	4
Refugee/migrant home in vicinity (1 = yes, 2016)	0.72	0.45	0	1
Direct contact with refugees/migrants (1 = yes, 2016)	0.65	0.48	0	1

General attitude towards immigration was measured in November 2015 with the following five items answered on four-point scales (strongly agree, somewhat agree, somewhat disagree, strongly disagree) [German: stimme voll und ganz zu, stimme eher zu, stimme eher nicht, stimme überhaupt nicht zu]: (1) Immigrants take jobs away from native born Germans [in German: Einwanderer nehmen den gebürtigen Deutschen die Arbeitsplätze weg]; (2) Immigrants generally help to fill jobs where there are shortages of workers [in German: Einwanderer helfen in der Regel, im Falle eines Arbeitskräftemangels Arbeitsplätze zu besetzen], (3) Immigrants help create jobs as they set up new businesses [in German: Einwanderer tragen zur Schaffung von Arbeitsplätzen bei, weil sie neue Unternehmen gründen]; (4) Immigrants bring down the wages of German citizens [in German: Durch Einwanderer sinken die Löhne von deutschen Staatsbürgern]; (5) Immigrants are a burden on the social system [in German: Einwanderer sind eine Belastung für das Sozialsystem]. Items (1) to (4) are taken from German Marshall Fund's Survey on Transatlantic Trends on Immigration4. Answers were coded in a way that higher values indicate more positive attitudes towards immigration. A factor analysis (n=434) with subsequent varimax rotation results in one factor with an Eigenvalue above 1, i.e. 2.86, 57% explained variance and factor loadings of at least 0.723. Cronbach's Alpha is 0.81. We constructed an additive index of the five items and divided the index scores by five. The mean of this index is 2.65, SD = 0.60, minimum = 1, maximum = 4.

Whether the respondents have a *refugee/migrant home in their vicinity* was measured with a single question in November 2016: "Is there a refugee/migrant home in your surroundings, within a radius of 2.500 m around your flat/house?" (Yes, No, Don't Know). [In German: "Gibt es in Ihrer Wohnumgebung, wir meinen damit einen Umkreis von bis zu 2500m um Ihr Haus/Ihre Wohnung, eine Unterkunft für Flüchtlinge und/oder Migranten?" (Ja, Nein, Weiss nicht)]

Direct contact with refugees/migrants was measured with the following question: “Have you ever had contact to a refugee/migrant?” (Yes, No) [In German: “ Hatten Sie schon einmal direkten Kontakt mit Flüchtlinge und/oder Migranten?” (Ja, Nein)]

As shown in Table B, an analysis of selection bias regarding retest participation using a binary logit model reveals a statistically significant and positive effect for age only. Older respondents have a slightly higher likelihood to participate in the retest survey. Most notably we do not find any significant effect of the attitude towards immigration on the likelihood of retest participation.

Table B. Binary logit model on retest participation

Variable (year)	b (z-value)
Sex (1 = women, 2015)	-.150 (-1.03)
Age in years (2015)	**.016 (3.08)**
Education in years (2015)	.0176 (0.81)
Attitude towards immigration (2015)	-.057 (-0.46)
Constant	.001 (0.00)
Mc-Fadden R^2	0.012
n	854

Notes: unstandardized coefficients; bold values indicate $p < 0.05$.

Experimental design

In the stated choice experiment respondents were asked to choose between three alternatives regarding migrant and refugee homes in their vicinity the alternative they prefer most. The choice alternatives were labeled “Refugee Home,” “Migrant Home,” and “Refugee and Migrant Home” representing whether refugees, migrants or a mix of both live in the planned homes. The order of these alternatives was randomized across choice sets. The choice tasks included five choice attributes (see below) with eight levels (country of origin and religion), six levels (number of persons), two levels (families or single persons), and four levels (type of home and distance to respondent’s house/flat). With respect to country of origin and religion we selected two countries (Syria and Serbia) that are within the top-ten countries in terms of numbers of asylum seekers being registered Germany in 2015, and two countries (Nigeria and India) that are less common as countries of origin of asylum seekers in Germany [1]. Another important selection criterion was that all of these countries have a sizeable population of Christians, which forms a common reference regarding religion.

The full factorial of all attribute-level combinations is 3,623,878,656 = (8 x 6 x 2 x 4 x 4) x (8 x 6 x 2 x 4 x 4) x (8 x 6 x 2 x 4 x 4). We employed an optimal orthogonal in the differenced (OOD) design [2]. Orthogonality ensures that the influence of a single attribute can be determined independently from the influences of the others. Besides orthogonality, the choice design was constructed to minimize the overlap between attribute levels across alternatives in a choice set, thus forcing respondents to make trade-offs between the single attributes. We obtained 36 choice sets which were blocked into six groups of six sets each, and each respondent answered one such group. The order of choice-sets within each group was randomized.

Description of experimental tasks

Instruction to the stated choice experiment in November 2015 and November 2016

In English

[Screen 1]

"New homes have to be established for refugees and migrants who come to Germany. **We would like to know which homes you would prefer.** On the following choice sets you can choose between different plans for the establishment of a new home. **Please imagine that these homes will be built in your place of residence and will be there for at least 3 years.**

The home can be for the accommodation of

- Refugees only or
- Migrants only or
- Refugees and Migrants.

Refugees are persons who were forced to flee (e.g. due to membership of a specific social group or their political beliefs). Migrants are persons who left their country voluntarily (e.g., because they hope for a better life). Both groups – refugees and migrants – can ask for asylum in Germany. Then they are asylum seekers. It is more likely for refugees than migrants that their asylum application will be confirmed and that they can stay in Germany. In principle there can come refugees and migrants from any country to Germany."

[Screen 2]

"In the following each home for refugees and/or migrants will be described with the following attributes.

Main country of origin and religion: The persons can be mainly from Syria (Muslims), Syria (Christians), Serbia (Serbian-Orthodox), Serbia (Christian), Nigeria (Muslims), Nigeria (Christian) or India (Hindus), India (Christians).

Number of persons: The home can accommodate 12, 32, 84, 125, 220, 350 persons.

Mainly families or single persons: The persons can be mainly families with children or single persons.

Type of home: The home can be an empty, renovated house; a container; an empty large building (e.g., building center or hospital) or an existing multi-purpose hall (e.g., gymnasium) can be used.

Distance to your house / flat: The home can be in distance of 500m, 1000m, 1700m or 2500m from your house/flat."

[Screen 3]: Example of a choice set

"The establishment of a home for refugees and/or migrants in your living environment could be as described in the choice sets. Please choose the best alternative for you. (Please assume that all alternatives would be feasible in your place of residence.)"

	Refugee Home	Refugee and Migrant Home	Migrant Home
Main country of origin and religion	Syria (Muslims)	Syria (Christians)	Serbia (Serbian-Orthodox)
Number of persons	220	350	12
Mainly families or single persons	Families with children	Families with children	Single persons
Type of home	Container building	Renovated house	Multi-purpose hall
Distance form your house/flat	1700m	2500m	500m
I choose …	□	□	□

In German

[Screen 1]

"Für Flüchtlinge und Migranten, die nach Deutschland kommen, müssen neue Unterkünfte eingerichtet werden. **Wir möchten wissen, welche Unterkünfte Sie persönlich bevorzugen würden.** Auf den folgenden Auswahl-Sets können Sie zwischen verschiedenen Vorhaben zur Einrichtung einer neuen Unterkunft für Flüchtlinge und Migranten wählen. **Stellen Sie sich vor, dass diese Unterkünfte in Ihren Wohnort gebaut werden sollen und für mindestens 3 Jahre bestehen bleiben.**

Die Unterkunft kann für die Unterbringung von

- ausschließlich Flüchtlingen oder
- ausschließlich Migranten oder
- von Flüchtlingen und Migranten genutzt werden.

Als Flüchtlinge werden Personen bezeichnet, die zur Flucht (z.B. wegen Zugehörigkeit zu einer bestimmten sozialen Gruppe oder ihrer politischen Überzeugungen) gezwungen wurden. Migranten

sind jene Personen, die ihr Land aus freiem Antrieb verlassen haben (z.B. weil sie sich ein besseres Leben erhoffen). Beide Gruppen – Flüchtlinge und Migranten – können in Deutschland einen Asylantrag stellen und gelten dann als Asylbewerber. Für Flüchtlinge ist es wahrscheinlicher als für Migranten, dass ihr Asylantrag positiv entschieden wird und sie in Deutschland bleiben können. Aus einem Land können prinzipiell sowohl Flüchtlinge als auch Migranten nach Deutschland kommen."

[Screen 2]

"Im Folgenden wird jede Unterkunft für Flüchtlinge und/oder Migranten wird durch folgende Merkmale beschrieben:

Hauptsächliches Herkunftsland und Religion: Die Personen stammen hauptsächlich aus Syrien (Muslime), Syrien (Christen), Serbien (Serbisch-Orthodoxe), Serbien (Christen), Nigeria (Muslime), Nigeria (Christen) oder Indien (Hindus), Indien (Christen).

Anzahl Personen: Die Unterkunft kann 12, 32, 84, 125, 220, 350 Personen aufnehmen.

Hauptsächlich Familien oder Einzelpersonen: Die Personen können hauptsächlich Familien mit Kindern sein oder Einzelpersonen.

Art der Unterkunft: Bei der Unterkunft kann es sich um ein leerstehendes, saniertes Wohnhaus; ein Containerbau; ein leerstehendes größeres Gebäude (z.B. Baumarkt oder Krankenhaus) oder die Nutzung einer bestehenden Mehrzweckhalle (z.B. Turnhalle) handeln.

Entfernung von Ihrem Haus / Ihrer Wohnung: Die Unterkunft kann von Ihrem Haus/Wohnung 500m, 1000m, 1700m oder 2500m entfernt liegen."

[Screen 3]: Example of a choice set

“Die Einrichtung einer Unterkunft für Flüchtlinge und/oder Migranten in ihrer Wohnumgebung könnte wie in den Alternativen im Auswahlset beschrieben erfolgen. Bitte wählen Sie die für Sie beste Alternative. (Bitte nehmen Sie an, dass alle Alternativen in ihrem Wohnort machbar sind.)”

	Unterkunft für Flüchtlinge	Unterkunft für Migranten	Unterkunft für Flüchtlinge und Migranten
Hauptsächliches Herkunftsland und Religion	Syrien (Muslime)	Syrien (Christen)	Serbien (Serbisch-Orthodoxe)
Anzahl Personen	220	350	12
Hauptsächlich Familien oder Einzelpersonen	Familien mit Kindern	Familien mit Kindern	Einzelpersonen
Art der Unterkunft	Containerbau	Saniertes Haus	Mehrzweckhalle
Entfernung von Ihrem Haus / ihrer Wohnung	1700m	2500m	500m
Ich wähle …	□	□	□

Statistical model

To understand citizens’ preferences towards new homes for refugees and migrants and to model transitions in these preferences over time, various models were estimated. In the following we discuss the modeling strategy and the results of selected models. All models were estimated using Latent Gold Choice 5.1 [3].

To obtain an initial impression of the preferences in the sample as a whole we started with the standard Multinomial Logit Model (MNL), in which we included respondents from both waves (n=418) and all attribute levels as described in the previous section. This model yielded a (pseudo) R^2 of 0.1538, indicating that the attributes could reasonably well predict individuals’ choices.

Table C presents the parameter estimates of the initial model. The results indicate that individuals prefer refugee homes and the combination of refugee and migrant homes over (exclusive) migrant homes. Compared to Syrian Muslims, individuals prefer Christian refugees/migrants, irrespective of the country of origin, while Muslims from Nigeria are least preferred. Individuals generally prefer less people being sheltered near their home and they prefer families over single persons. With regard to the type of home, an empty large building and renovated house are both preferred over a multi-purpose hall or a container building. Finally, individuals prefer larger distances between their own house and the sheltering location. Overall, the results indicate that individuals (taken as a whole) are rather negative towards sheltering refugees and migrants near their home.

Table C. Parameter estimates of the baseline MNL model

**Attribute**	**Attribute level**	**Estimate**	**SE**
Intercepts (ref.= Refugee and Migrant homes)	Refugee homes	**0.0862**	0.039
	Migrant homes	**-0.3308**	0.044
Main country of origin and religion (ref.= Syria (Muslims))	India (Christians)	**0.3478**	0.076
	India (Hindus)	-0.0309	0.085
	Nigeria (Christian)	*0.1590*	0.087
	Nigeria (Muslims)	**-0.3735**	0.097
	Serbia (Christian)	**0.2611**	0.091
	Serbia (Serbian-Orthodox)	*-0.1602*	0.083
	Syria (Christians)	**0.5481**	0.071
Number of persons (/ 100)	Continuous	**-0.1944**	0.019
Type of migrants/refugees (ref.=single person)	Mainly families	**0.8023**	0.036
Type of home (ref.= a container)	An empty large building	**0.6487**	0.052
	Multi-purpose hall	0.0376	0.054
	Renovated house	**0.7547**	0.053
Distance to your house / flat (in kilometers)	Continuous	**0.0918**	0.027
**log-likelihood**	-4396.1		
**Number of parameters**	15		
**R^2**	0.1538		

SE = standard error
Estimates in bold are significant at p<0.05
Estimates in italic are significant at p<0.10

Since our interest was not in revealing individuals' preferences towards a range of countries and religions, but was actually focused on preferences towards Muslim and Syrian migrants/refugees, we estimated a second MNL model in which we only considered these attribute levels. Therefore, new dummy variables were created for Muslims (from either Syria or Nigeria) and for Syria (including Muslims and Christian). The other religions and countries were defined as the reference group. Re-estimation of this – considerably more parsimonious – MNL model yielded a pseudo R^2 of 0.1474, indicating that the explanatory power of the model decreased only marginally. It should be noted, however, that the fit of the model (in terms of the Log-likelihood) was significantly lower.

Table D ("Waves 2015/2016") presents the parameter estimates of the second model. While most parameter estimates remain more or less the same (and are therefore not discussed a second time), the estimates relating to the country and religion variable indicate that, compared to the other countries and religions, Muslims are preferred less, while refugees/migrants from Syria are preferred more. Hence, there seems to be a clear anti-Muslim sentiment in the population. Regarding the preference towards Syrians, a plausible explanation may be that individuals believe that people fleeing from this country represent 'true' refugees. Table D ("Full sample 2015") further indicates that there is no selection bias with respect to retest participation because the results for the full sample are similar to the results of the retest sample.

Table D. Parameter estimates of the MNL model considering Syria and Muslims

		Waves 2015/2016 (n=418)		Full sample 2015 (n=836)	
**Attribute**	**Attribute level**	**Estimate**	**SE**	**Estimate**	**SE**
Intercepts (ref.= Refugee and Migrant homes)	Refugee homes	**0.0907**	0.039	**0.1876**	0.062
	Migrant homes	**-0.2767**	0.042	**-0.2405**	0.068
Main country of origin and religion (ref.= all other)	Muslim	**-0.5308**	0.046	**-0.4402**	0.072
	Syrian	**0.4528**	0.053	**0.6119**	0.085
Number of persons (/ 100)	Continuous	**-0.2233**	0.018	**-0.1864**	0.029
Family (ref.=single person)	Mainly families	**0.7839**	0.035	**0.7185**	0.057
Type of home (ref.= a container)	An empty large building	**0.6066**	0.049	**0.6014**	0.078
	Multi-purpose hall	0.0012	0.053	-0.0410	0.085
	Renovated house	**0.7113**	0.051	**0.6053**	0.081
Distance to your house / flat (in kilometers)	Continuous	**0.1424**	0.025	**0.1674**	0.040
**Log-likelihood**		-4427.40		-1722.27	
**Number of parameters**		10		10	
**R^2**		0.1474		0.1369	

SE = standard error
Estimates in bold are significant at $p<0.05$

We applied latent class choice modelling in order to reveal and explain transitions in preferences over time. Typically, the decision to consider a certain number of latent classes is based on model fit and model parsimony (i.e. the number of parameters). Various Information Criteria (weighing both) are available to this end [4]. In the context of latent class modelling, the Bayesian Information Criterion has been shown to perform particularly well and is therefore considered in this analysis [5].

Modelling individuals' transitions between the latent classes over time requires a low error rate in the modal assignment of individuals, i.e. the assignment to a latent class based on the highest probability. Otherwise, observed transitions between the latent classes over time could not be (solely) attributed to real preferences shifts, but would instead also reflect measurement errors. Therefore, as a second criterion in deciding upon the number of latent classes, the proportion of classification errors was considered [6].

Using the model considering only Syria and Muslims as country/religion (Table D "Waves 2015/2016") as the baseline '1-class' model, Table E presents the model fit (LL and R^2), the BIC values and the proportions of classification errors of models with 2-5 latent classes. The results indicate that the BIC value keeps decreasing with increasing numbers of latent classes, indicating that, based on this criterion, the optimal model is one with at least 5 classes. Such a high number could not be reasonably handled in the transition model. Hence, the BIC criterion was not useful in deciding on the number of classes.

In terms of the proportion of classification errors the 2-class model, with an error rate of around 8%, performed much better than models with 3-5 classes, with an error rate of around 16-17%. It follows

that based on this criterion the 2-class model was clearly preferable for the purpose of our analysis. The decision was therefore made to opt for the parsimonious 2-class model. Selection of this model also meant that the complexity of the transition model would be kept at a relatively low level, and hence, that this model could be interpreted in a straightforward way.

Table E. Model fit and proportion of classification errors of LC models

**Model description**	**LL**	**Npar**	**BIC(LL)**	**Class. Err.**	**R^2**
1-Class MNL (Table 2)	-4427.4	10	8922.1	0.0000	0.1474
2-Class MNL	-4296.6	27	8774.9	0.0789	0.2262
3-Class MNL	-4200.2	44	8696.4	0.1631	0.3293
4-Class MNL	-4112.6	61	8635.6	0.1744	0.3924
5-Class MNL	-4037.9	78	8600.7	0.1591	0.4192

LL = log-likelihood of the final model
Npar = Number of parameters
BIC(LL) = Bayesian Information Criterion (based on LL)
Class. Err. = Proportion of classification errors

Table F presents the parameter estimates of the 2-class LC model. Approximately 80% of the sample is assigned to the first class. Given this large share it is logical that the parameter estimates of this class strongly resemble the estimates of the 1-class MNL model (Table D). However, across all estimates there is a consistent difference towards a stronger rather disapproving preference. For example, there is a stronger dislike of (exclusive) migrant homes and Muslims. In addition, individuals' choices in class 1 are more strongly influenced by the number of people being sheltered and the distance between their home and the shelter location.

Citizens in class 2, representing the remaining 20%, have a more positive preference for refugee homes. They are indifferent as to whether refugees, migrants of a combination of both are being sheltered near their home and are also indifferent with regard to the number of refugees being sheltered. Similar to class 1 they prefer Syrian people and families (over single persons), but they have no particular dislike of Muslims. Individuals in class 2 have a strong preference for the two higher quality accommodations – an empty large building and renovated house – over the two less quality accommodations – a multi-purpose hall and container. Finally, the sign of the variable 'distance to you house / flat' is opposite to the sign in the first class, indicating that individuals in class 2 actually prefer the sheltering location to be closer to their home.

The (pseudo) R-square of the 2-class model (R^2=0.2262) is substantially higher than the 1-class model (R^2=0.1474). Interestingly, this increase can mainly be attributed to the second class, in which the choices can be predicted very well by the included attributes (class 2 R^2=0.4286).

Finally, the (significant) influences of the six variables in the class membership model are in line with the preferences of both classes: individuals who are higher educated, have a stronger general pro-immigrant attitude, who have been in contact with refugees, and already have a sheltering location

near one's home (significant at 10%) have a significantly higher probability to belong to the second class. Gender and age do not significantly influence class membership.

Table F. Parameter estimates of the 2-class choice model

		Class 1		Class 2	
**Class size**		0.7966		0.2034	
**Attribute**	**Attribute levels**	**Estimate**	**SE**	**Estimate**	**SE**
Intercepts (ref.= Refugee and Migrant homes)	Refugee homes	0.0821	0.045	0.1474	0.132
	Migrant homes	**-0.4062**	0.051	-0.1314	0.234
Main country of origin and religion (ref.= all other)	Muslim	**-0.7347**	0.061	0.2030	0.183
	Syrian	**0.4836**	0.062	**0.4512**	0.200
Number of persons (/ 100)	Continuous	**-0.2618**	0.023	-0.0932	0.064
Family (ref.=single person)	Mainly families	**0.8535**	0.045	**0.7970**	0.154
Type of home (ref.= a container)	An empty large building	**0.3187**	0.067	**2.2217**	0.317
	Multi-purpose hall	-0.0370	0.060	0.2549	0.314
	Renovated house	**0.2719**	0.071	**2.7609**	0.346
Distance to your house / flat (in kilometers)	Continuous	**0.2264**	0.032	**-0.2488**	0.091
**Class membership (ref.=class 2)**		**Estimate**	**SE**		
Intercept		**9.2817**	1.542		
Gender (ref.=male)	Female	0.3197	0.288		
Age	Continuous	-0.0002	0.010		
Education level	Continuous	**-0.0873**	0.040		
Pro-immigrant attitude	Continuous	**-1.9987**	0.362		
Contact (ref.=no)	Yes	**-0.8110**	0.352		
Shelter near home (ref.=no)	Yes	*-0.6781*	0.387		
	**Pseudo-R^2**				
**Class 1**	0.1426				
**Class 2**	0.4286				
**Overall**	0.2262				

SE = standard error

Estimates in bold are significant at p<0.05

Estimates in italic are significant at p<0.10

The model results reported in Table F are used to investigate how changes in the specification of a refugee or migrant home affect the probability of choosing this home. Changes in the choice probability are calculated based on a choice between three homes, using a sequential process where one characteristic of one out of the three refugee/migrant homes is altered at a time, while the remaining two homes retain a reference specification. This reference home is defined by the following characteristics: joint accommodation of refugees and migrants; non-Syrians; Muslims; mainly single persons; container building; accommodation of 138 people (mean of attribute levels 'Number of persons'); distance to respondents' home 1.43km (mean of attribute levels 'Distance to your home/flat'). Multinomial logit choice probabilities are calculated using parameter estimates for each of the two classes. If all three homes share the same (reference) specification, the probability of

choosing any one out of the three homes is 1/3. If the specification of one home is altered, for example if the home accommodates Syrians rather than non-Syrians or Muslims rather than non-Muslims, the choice probability of this home will increase or decrease relative to the probability of choosing any of the two reference homes. The magnitude of the difference in choice probability between the evaluated home compared to the reference homes illustrates the relative influence of the different characteristics on respondents' decisions. Table G below reports the probabilities for selected characteristics. 95% confidence intervals for differences in probability between evaluated and reference home are calculated using a Krinsky and Robb [7] procedure.

Table G. Choice probabilities relative to reference specification for the 2-class choice model

**Changes evaluated**	**Class**	**Probability of choosing evaluated home (%)**	**Probability of choosing either of two reference homes (%)**	**Difference in probability between evaluated and reference home (%)**	**95% confidence intervals for difference in probability (2.5%; 97.5%)**
Syrians (vs. non-Syrians)	1	44.8	27.6	17.2	12.6;21.9
	2	44	28.0	16.0	1.5;29.4
Muslims (vs. non-Muslims)	1	19.3	40.3	-21.0	-23.7;-18.1
	2	38	31.0	7.0#	-4.8;19.5
Families (vs. single persons)	1	54.0	23	31.0	27.8;34.2
	2	52.6	23.7	28.9	17.9;40.6
Empty large building (vs. container)	1	40.75	29.6	11.1	6.4;15.9
	2	82.2	8.9	73.3	56.4;84.1
Multi-purpose hall (vs. container)	1	32.5	33.7	-1.2#	-5.2;2.6
	2	39.2	30.4	8.8#	-12.0;31.2
Renovated house (vs. container)	1	39.6	30.2	9.4	4.4;14.3
	2	88.8	5.6	83.2	69.2;90.9
350 people (max) (vs. 138 people)	1	22.3	38.8	-16.5	-18.9;-14.1
	2	29.1	35.4	-6.4#	-14;2.5
500 m distance to house (min) (rather than 1.43km)	1	28.8	35.6	-6.8	-8.4;-5
	2	38.7	30.7	8.0	2.4;14.0

Notes: # indicates that effect was not significantly different from zero in latent class choice model; corresponding effects on probabilities are also not significantly different from zero. Class 1 refers to the "rather disapproving" class and Class 2 to the "rather approving" class.

As stated above, the identification of the two classes allowed modelling and interpreting transitions of individuals between these classes over time. To this end, a 2-state Markov model was estimated [8]. Based on modal assignment, each individual in each wave is (deterministically) assigned to one of the two classes, i.e. the class with highest membership probability for that individual. In the Markov model it is assumed that class membership represents a 'state' of an individual. In line with the first-order Markov assumption, it is assumed that a person's state membership at the second point in time (2016) is influenced by his/her state membership at the first point in time (2015). In addition, the six explanatory variables previously included in the class membership function are again included in the model and assumed to predict initial state membership as well as state membership at the second point in time.

In order to test for parameter equality we compared constrained (MNL) models where equality constraints were imposed on specific model parameters with the baseline MNL model. As shown below in Tables H and I, likelihood ratio tests indicate that in all cases the models with equality constraints imposed on the parameters (with more degrees of freedom) result in significant reductions in model fit. Hence, the (absolute values of the) parameters associated with "mainly families," "an empty large building" and "multi-purpose hall" are significantly different and greater than the parameters associated with "Syria" and "Muslims."

Table H. Baseline MNL model for testing parameter equality

**Attribute**	**Attribute level**	**Estimate**	**SE**
Intercepts (ref.= Refugee and Migrant homes)	Refugee homes	**0.0907**	0.039
	Migrant homes	**-0.2767**	0.042
Main country of origin and religion (ref.= all other)	Muslims (a)	**-0.5308**	0.046
	Syrian (b)	**0.4528**	0.053
Number of persons (/ 100)	Continuous	**-0.2233**	0.018
Family (ref.=single person)	Mainly families (c)	**0.7839**	0.035
Type of home (ref.= a container)	An empty large building (d)	**0.6066**	0.049
	Multi-purpose hall	0.0012	0.053
	Renovated house (e)	**0.7113**	0.051
Distance to your house / flat (in kilometers)	Continuous	**0.1424**	0.025
**Log-likelihood**	-4427.4		
**Number of parameters**	10		
**R^2**	0.1474		

Table I. Constrained MNL models in comparison to the baseline MNL model

**Model description**	**LL**	**BIC(LL)**	**Npar**	**R²**	**2*LLdif**	**p-value**
MNL (only Syrian and Muslim)	-4427.3983	8922.083	10	0.1474		
MNL (a=-c)	-4436.9671	8934.492	9	0.1448	19.1376	1.21625E-05
MNL (a=-d)	-4454.0697	8968.697	9	0.1432	53.3428	2.80136E-13
MNL (a=-e)	-4430.6252	8921.808	9	0.1467	6.4538	0.011071529
MNL (b=c)	-4440.2473	8941.052	9	0.1440	25.698	3.99241E-07
MNL (b=d)	-4445.1749	8950.908	9	0.1442	35.5532	2.48179E-09
MNL (b=e)	-4434.4946	8929.547	9	0.1459	14.1926	0.000165018

Table J presents the parameter estimates of the 2-state Markov model. The estimates related to initial state membership resemble those of the membership model in the 2-class choice model (Table F). However, it should be noted that, due to the modal assignment of individuals (via which measurement errors are introduced), education level is no longer significant. The probability of being member of the first state in 2016 is positively influenced by state-1 membership in 2015. This means that, irrespective of (i.e., also when controlling for) the influences of the included explanatory variables, there is an 'autonomous' tendency of citizens to stay in or move to the first state. Finally, education level, the pro-immigrant attitude and contact with refugees decrease the probability of staying in / moving to state 1 in 2016.

Table J. Parameter estimates of the 2-state Markov model.

**State membership (ref.=state 2) in 2015**		**Estimate**	**SE**
Intercept		**11.9159**	1.553
Gender (ref.=male)	Female	0.4192	0.320
Age	Continuous	0.0094	0.011
Education level	Continuous	-0.0662	0.046
Pro-immigrant attitude	Continuous	**-2.8878**	0.401
Contact (ref.=no)	Yes	**-1.0779**	0.399
Shelter near home (ref.=no)	Yes	**-0.8934**	0.412
**State membership (ref.=state 2) in 2016**			
Intercept		**9.5530**	1.623
State in 2015 (ref.=state 2)	State 1	**1.1904**	0.352
Gender (ref.=male)	Female	0.5028	0.319
Age	Continuous	-0.0062	0.011
Education level	Continuous	**-0.1475**	0.046
Pro-immigrant attitude	Continuous	**-1.9265**	0.389
Contact (ref.=no)	Yes	**-0.9478**	0.388
Shelter near home (ref.=no)	Yes	*-0.7062*	0.406

SE = standard error
Estimates in bold are significant at p<0.05
Estimates in italic are significant at p<0.10

The parameter estimates can be used to compute the predicted transition probabilities reflecting the movement of individuals across the two waves. Table K presents this matrix. Individuals assigned to state 1 (rather disapproving) in 2015 have a probability of 90% of remaining in this state in 2016. In contrast, citizens assigned to state 2 (rather approving) only have a probability of 56% of remaining in their respective state in 2016. They have a substantial probability (44%) of moving from the "rather approving" state to the "rather disapproving" state.

Table K. Matrix of transition probabilities

**State in 2015**	**State in 2016**	
	**1**	**2**
**1**	0.8986	0.1014
**2**	0.4371	0.5629

References

1. BAMF. *Aktuelle Zahlen zu Asyl* http://www.bamf.de/SharedDocs/Anlagen/DE/Downloads/Infothek/Statistik/Asyl/aktuelle-zahlen-zu-asyl-dezember-2015.html?nn=795222 (2015).
2. Burgess, L. & Street, D. J. Optimal designs for choice experiments with asymmetric attributes. *Journal of Statistical Planning and Inference* ***134*****,** 288-301 (2005).
3. Vermunt, J. K. & Magidson, J. *Technical guide for Latent GOLD Choice 4.0: basic and advanced.* (Belmont: Statistical Innovations Inc., 2005).
4. Akaike, H. Likelihood of a model and information criteria. *Journal of econometrics* **16,** 3-14 (1981).
5. Nylund, K. L., Asparouhov, T. & Muthén, B. O. Deciding on the number of classes in latent class analysis and growth mixture modeling: A Monte Carlo simulation study. *Structural equation modeling* **14,** 535-569 (2007).
6. Johnston, R. J., Boyle, K. J., Adamowicz, W., Bennett, J., Brouwer, R., Cameron,T. A., Hanemann,W.M., Hanley, N., Ryan, M., Scarpa, R., Tourangeau, R., & Vossler, C. A.. Contemporary Guidance for Stated Preference Studies. Journal of the Association of Environmental and Resource Economists, 4, 319–405 (2017).
7. Krinsky, I. & Robb, A. L. On Approximating the Statistical Properties of Elasticities. Review of Economics and Statistics 68(4), 715–719 (1986).
8. Vermunt, J. K., Tran, B. & Magidson, J. Latent class models in longitudinal research. In Menard, S. (ed.), *Handbook of longitudinal research: Design, measurement, and analysis* 373-385 (Cambridge: Academic Press, 2008).
